# Supplementary figures and images for: A Role for Human Renal Tubular Epithelial Cells in Direct Allo-Recognition by CD4+ T-Cells and the Effect of Ischemia-Reperfusion
Source: Int J Mol Sci. 2021 Feb 9;22(4):1733. doi: 10.3390/ijms22041733 (PMC7915934; doi:10.3390/ijms22041733)

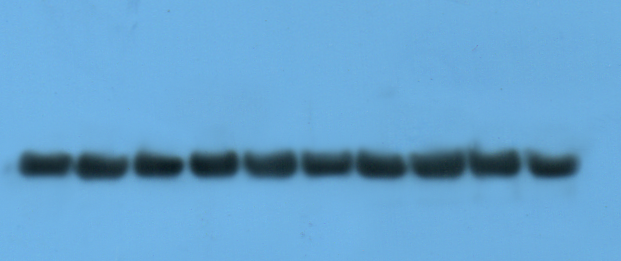

Supplement: Supplementary file 1 [file ijms-22-01733-s001.zip › Original WB bands/Actin in fig 2A.tif]

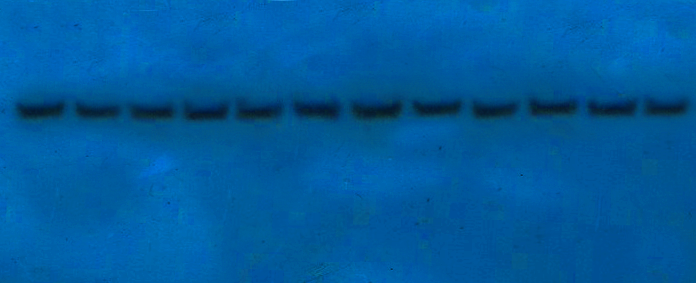

Supplement: Supplementary file 1 [file ijms-22-01733-s001.zip › Original WB bands/Actin in fig 3A.tif]

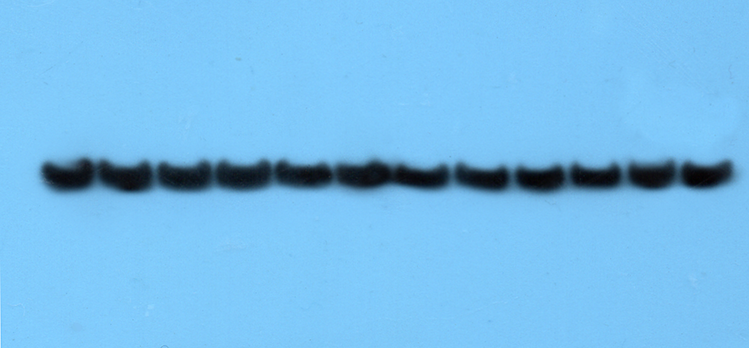

Supplement: Supplementary file 1 [file ijms-22-01733-s001.zip › Original WB bands/Actin in fig 4B.tif]

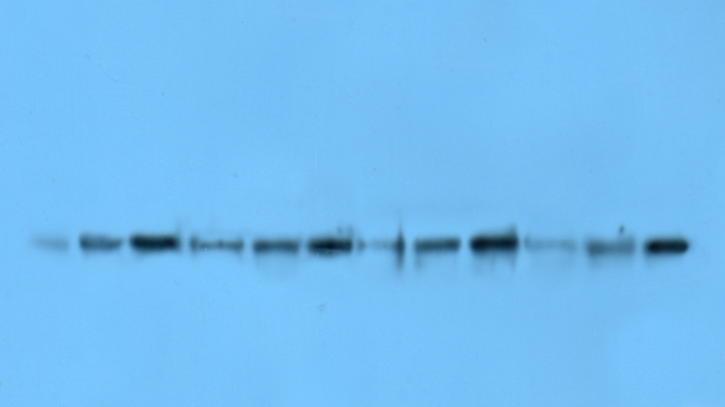

Supplement: Supplementary file 1 [file ijms-22-01733-s001.zip › Original WB bands/c-myc in fig 3A.tif]

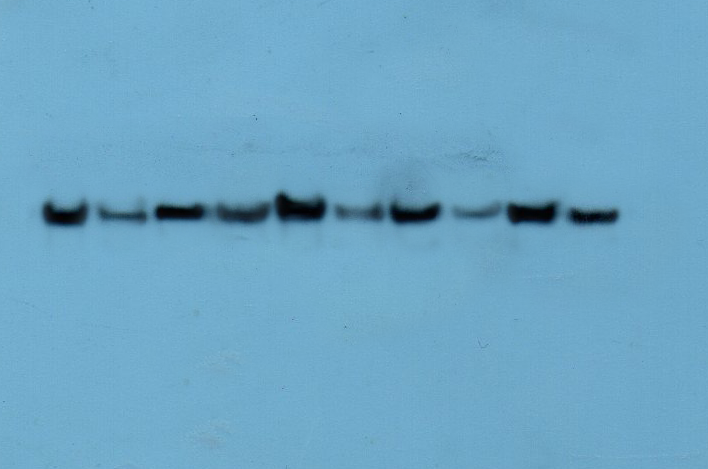

Supplement: Supplementary file 1 [file ijms-22-01733-s001.zip › Original WB bands/CD80 in fig 2A.tif]

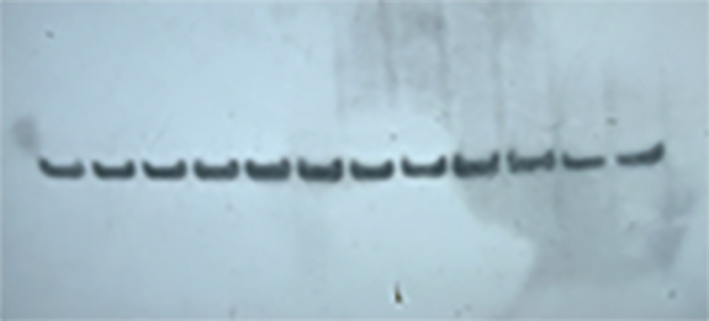

Supplement: Supplementary file 1 [file ijms-22-01733-s001.zip › Original WB bands/FoxP3 in fig 4B.tif]

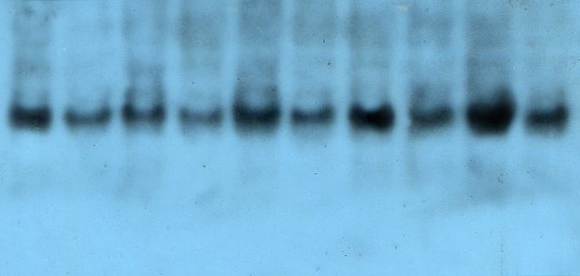

Supplement: Supplementary file 1 [file ijms-22-01733-s001.zip › Original WB bands/HLA-DR in fig 2A.tif]

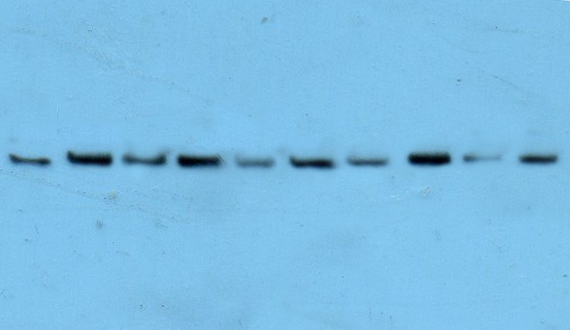

Supplement: Supplementary file 1 [file ijms-22-01733-s001.zip › Original WB bands/ICAM in fig 2A.tif]

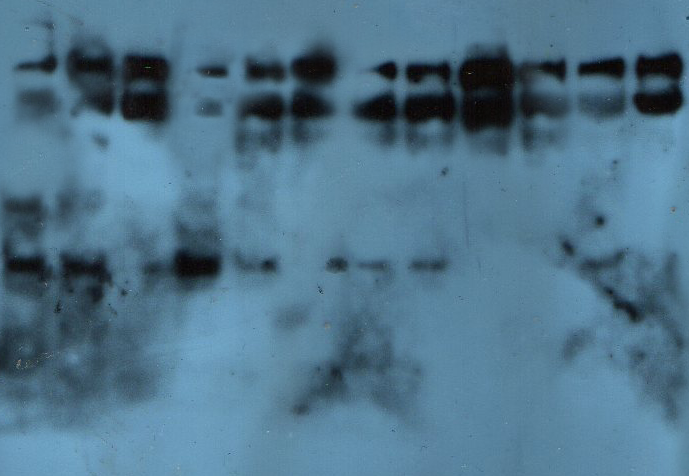

Supplement: Supplementary file 1 [file ijms-22-01733-s001.zip › Original WB bands/ki67 in fig 4B.tif]

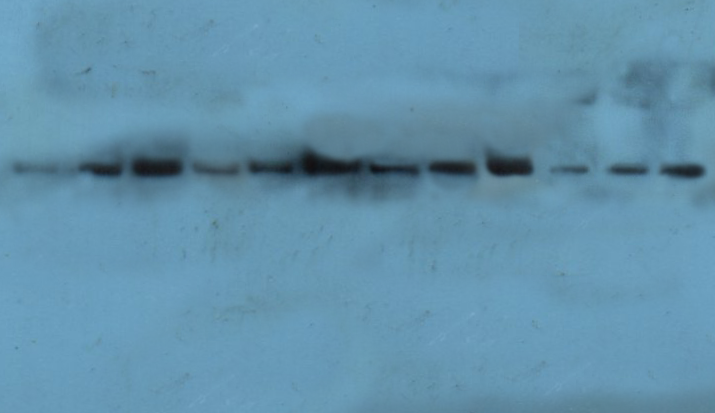

Supplement: Supplementary file 1 [file ijms-22-01733-s001.zip › Original WB bands/p-zeta in fig 3A.tif]

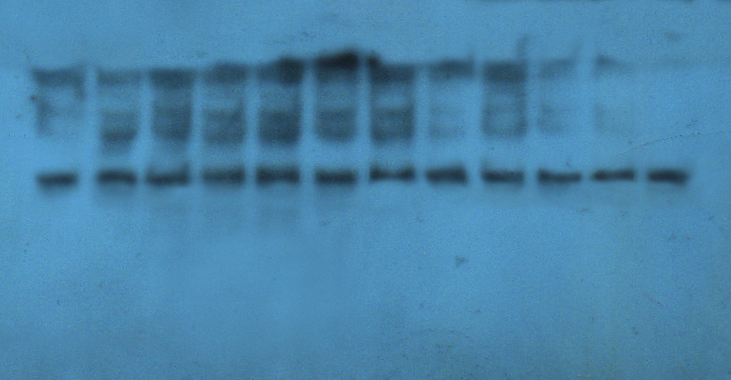

Supplement: Supplementary file 1 [file ijms-22-01733-s001.zip › Original WB bands/zeta in fig 3A.tif]
